# Systemic physiology augmented functional near-infrared spectroscopy hyperscanning: a first evaluation investigating entrainment of spontaneous activity of brain and body physiology between subjects

**DOI:** 10.1117/1.NPh.9.2.026601

**Published:** 2022-04-18

**Authors:** Sabino Guglielmini, Gino Bopp, Valentine L. Marcar, Felix Scholkmann, Martin Wolf

**Affiliations:** a University of Zurich, University Hospital Zurich, Department of Neonatology, Biomedical Optics Research Laboratory, Zurich, Switzerland; b University Hospital Zürich, Comprehensive Cancer Center Zürich, Zürich, Switzerland; c University of Bern, Institute of Complementary and Integrative Medicine, Bern, Switzerland

**Keywords:** hyperscanning, functional near-infrared spectroscopy, interpersonal brain synchronization, eye contact, cross-brain coherence, systemic physiology augmented functional near-infrared spectroscopy hyperscanning, systemic physiology augmented functional near-infrared spectroscopy, SPA-fNIRS

## Abstract

**Significance:**

Functional near-infrared spectroscopy (fNIRS) enables measuring the brain activity of two subjects while they interact, i.e., the hyperscanning approach.

**Aim:**

In our exploratory study, we extended classical fNIRS hyperscanning by adding systemic physiological measures to obtain systemic physiology augmented fNIRS (SPA-fNIRS) hyperscanning while blocking and not blocking the visual communication between the subjects. This approach enables access brain-to-brain, brain-to-body, and body-to-body coupling between the subjects simultaneously.

**Approach:**

Twenty-four pairs of subjects participated in the experiment. The paradigm consisted of two subjects that sat in front of each other and had their eyes closed for 10 min, followed by a phase of 10 min where they made eye contact. Brain and body activity was measured continuously by SPA-fNIRS.

**Results:**

Our study shows that making eye contact for a prolonged time causes significant changes in brain-to-brain, brain-to-body, and body-to-body coupling, indicating that eye contact is followed by entrainment of the physiology between subjects. Subjects that knew each other generally showed a larger trend to change between the two conditions.

**Conclusions:**

The main point of this study is to introduce a new framework to investigate brain-to-brain, body-to-body, and brain-to-body coupling through a simple social experimental paradigm. The study revealed that eye contact leads to significant synchronization of spontaneous activity of the brain and body physiology. Our study is the first that employed the SPA-fNIRS approach and showed its usefulness to investigate complex interpersonal physiological changes.

## 1 Introduction

Humans are a social species that engage in complex interactions during goal-oriented cooperation.^1^ Social cognition is the basis for such interactions and includes three main components: simulation, empathy, and mentalization. The standard simulation notion refers to a functional process in which an observer tries to voluntarily reproduce, even with the help of imagination, the same mental states of another individual.^2^ First, social cognition has been attributed by Gallese^3^ to an embodied simulation enabling an immediate understanding and associated with mirror neuron systems, i.e., neuronal systems that are activated when an intentional action is performed (such as motor action) and when the observing the same action. Studies have shown activation of the motor cortex in 6-month-old children when observing actions.^4^^,^^5^ The second component is empathy, which is defined as the ability to share feelings and emotions.^6^ It is automatic, different for everyone and it is stronger depending on the type of relationship one has with the observed person.^7^^,^^8^ Third, mentalization is an essential part of social cognition and the ability to read the state of mind, such as desires, beliefs, and intentions in others.^9^^–^^11^

Past research has focused on establishing the link between behavior and brain activity of single subjects outside of real social interactions.^12^^–^^14^ Subsequent research studied individual brain activity during simulated social situations.^15^ Most studies about social cognition were performed considering an interpersonal relationship only from a purely social point of view. Some time ago it was already pointed out that the neurobiology of human social behavior is an important, but neglected research topic that requires more attention than it has attracted so far.^16^ Today it is possible to measure brain activity of two interacting subjects simultaneously, a technique referred to as hyperscanning.^17^^–^^23^ It is a well-established methodology for studying individuals during natural interactions^24^ and has been used in various contexts over a number of years. It allows monitor of both emotional^25^^,^^26^ and cognitive aspects^27^^–^^29^ of social interaction.

Several neuroimaging techniques have been adopted for hyperscanning, each with its advantages and disadvantages. Hyperscanning experiments were originally performed with electroencephalography (EEG) and it is still the technique that is used the most.^30^^–^^34^ While functional magnetic resonance imaging (fMRI) is probably the most prominent neuroimaging technology, it requires the subjects to enter a highly constrained space in the scanner and does not allow investigating social interactions in a naturalistic environment. The scanner generates loud noise likely to entrain rhythms in any physiological parameter. In contrast, functional near-infrared spectroscopy (fNIRS) is a noninvasive optical neuroimaging method that enables cortical brain activity to be measured in everyday situations and natural settings.^35^^,^^36^ fNIRS indirectly measures brain activity based on optical absorption changes in the brain tissue associated with changes in cerebral tissue oxygenation and hemodynamics, i.e., concentration changes of oxyhemoglobin ($\left[O_{2}\mathrm{Hb}\right]$), deoxyhemoglobin ([HHb]), and total hemoglobin ([tHb]).^37^ Our research group was one of the first to demonstrate the feasibility to use fNIRS for human hyperscanning studies.^27^^,^^38^^,^^39^

As demonstrated by Hamilton,^40^ properly studying social interactions “requires studying the brain and bodily coordination together” since “interacting brains exist within interacting bodies.” We adopted this concept. The measurement of brain-to-brain coupling needs to be accompanied by the measurement of body-to-body coupling and body-to-brain coupling. In our view, this is important for two reasons. First, it allows investigating intrasubject (within a subject) as well as intersubject (between subjects) body–brain interactions. Second, it enables to investigate how much systemic physiology impacts the fNIRS signals measured on the head. This is of particular importance since parameters measured by fNIRS are influenced by systemic physiology,^41^^,^^42^ e.g., changes in blood pressure^43^^,^^44^ and the concentration of ${\mathrm{CO}}_{2}$ in the blood.^45^^,^^46^ We investigated this aspect in detail in the last years and developed the methodology appropriate to measure and analyze fNIRS signals along with systemic physiological signals, which we termed “systemic physiology augmented fNIRS” (SPA-fNIRS).^47^^–^^49^ This approach has not yet been applied to hyperscanning which allows the introduction of a new methodology: SPA-fNIRS hyperscanning.

In our exploratory study, the primary interest was to propose a new framework enabling the investigation of interbody and interpersonal neural synchrony in any form of interaction between pairs of subjects. We employed SPA-fNIRS hyperscanning to capture intersubject brain-to-brain, brain-to-body, and body-to-body coupling during goal-oriented interaction while blocking and not blocking the visual communication between the subjects. To this end, the differences in the coupling strength were compared between a resting-state condition where the subjects were not looking at each other, and a condition where they were looking at each other. We hypothesized that the coupling of the signals between pairs is higher when they make eye contact compared with the condition when the visual communication channel is blocked.

## 2 Materials and Methods

### 2.1 Participants

27 pairs of healthy adults (mean age: 32.1 years, age range: 18 to 65 years, 24 males, and 30 females) participated in the study. None of them reported neurological, psychiatric or other diseases that might affect the results of our study. Due to the lack of optical coupling between the optodes caused by the dense hair of two participants, two pairs were excluded from the analysis. In addition, another dataset was excluded because the electrocardiogram electrodes in one participant were disconnected during the measurement. Thus, from 27 pairs, 24 were considered in the analysis. This study was approved by the Ethics Committee of the County of Zurich (ethics approval code KEK-ZH-Nr. E50/2002). All participants were informed about the experimental procedure and provided their informed, written consent. All participants were renumerated for participating in the study. The relation between the two individuals was assessed and categorized as familiar (siblings, spouses and boyfriends/girlfriends) or unfamiliar. The familiar group consisted of eight pairs of siblings and six couples (spouses and boy/girlfriends), while the unfamiliar group was composed of five colleagues pairs and five strangers pairs. The new guidelines for conducting and reporting fNIRS studies were taken into account.^50^

### 2.2 Data Acquisition and Experimental Paradigm

**Fig. 1:**
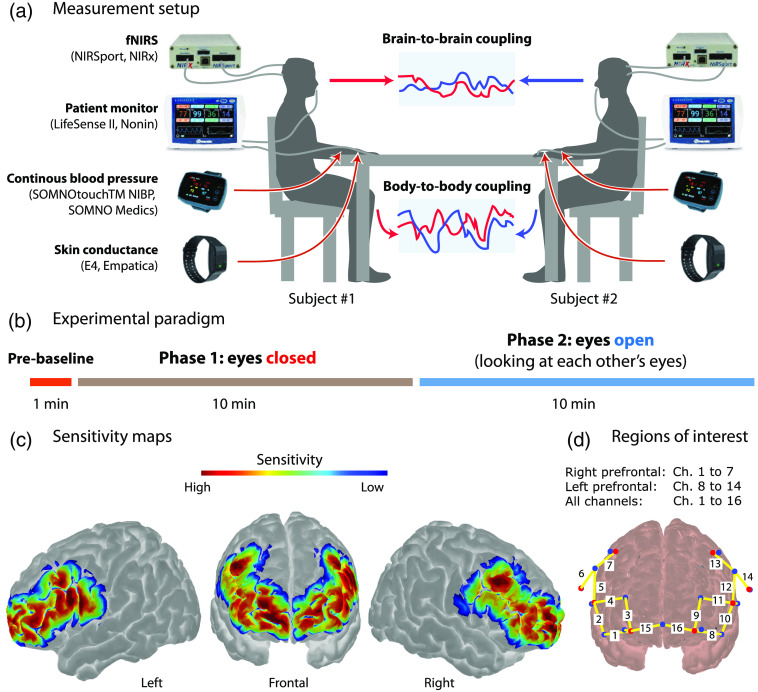
Experimental design. (a) Measurement setup. Participants sit in front of each other; (b) the two task conditions and the timeline for each experiment; (c) sensitivity maps generated with the Monte Carlo photon transport software for the depicted channel arrangement; (d) probe with the legend of the three ROIs.

Changes in $\left[O_{2}\mathrm{Hb}\right]$, [HHb], and [tHb] were measured on the head by optical neuroimaging with fNIRS (NIRSport, Berlin, Germany). The system provided 16-long-distance channels (30 mm), and 8-short-distance channels (8 mm) using the two wavelengths (760 and 850 nm). The sampling frequency was set to 7.81 Hz. Optodes were positioned according to the 10-20 system using individually sized caps (NIRx Medizintechnik GmbH, Berlin, Germany). Figure 1(c) depicts the sensitivity map highlighting the ability of the setup to measure the prefrontal cortex as well as the left and right frontotemporal cortex. The short distance channels were employed to capture changes in the superficial (extracerebral) tissue compartment.^51^^,^^52^

In addition, systemic physiology was measured by the following 11 parameters: heart rate (HR), mean arterial blood pressure (MAP), pulse pressure (PP), systolic blood pressure (SBP), diastolic blood pressure (DBP), arterial oxygenation (${\mathrm{SpO}}_{2}$), electrodermal activity (EDA), skin temperature (Temp) on the left and right wrist, and end-tidal carbon dioxide ($P_{\mathrm{ET}}{\mathrm{CO}}_{2}$). HR, MAP, PP, SBP, DBP, and ${\mathrm{SpO}}_{2}$ were acquired with the SOMNOtouchTM NIBP device (SOMNO Medics, Germany) at a sampling rate of 4 Hz, EDA and skin temperature with the E4 wristbands (Empatica, Boston, Massachusetts, United States) at 4 Hz, and $P_{\mathrm{ET}}{\mathrm{CO}}_{2}$ with a capnograph (Nonin Life Sense; Nonin Medical, Plymouth, Minnesota, United States) at 1 Hz. Figure 1(a) shows the measurement setup.

Paired participants were instructed to adopt comfortable posture on a chair and rested their heads on the backrest to reduce head movement. They were seated in front of each other on opposite sides of a table [Fig. 1(a)] at a distance of 180 cm.

A measurement session lasted 21 min. The experimental protocol was divided into two phases. In the first phase, no interaction between the subjects took place and they kept their eyes closed for 11 min. In the second phase, they looked at each other for 10 min. The first minute of the measurement (prebaseline) was discarded from the analysis with the aim of ensuring that the participants were settled and the measurement free of initial, task-unrelated changes [Fig. 1(b)].

Given the difficulty of maintaining eye contact for the entire duration of the second phase, participants were instructed to maintain eye contact for as long as possible and, in case of discomfort, to look at the other participant’s body and then focus on his/her eyes again. During the experiment, pairs were asked not to talk and minimize the movement of their head to prevent motion artifacts.

### 2.3 Signal Preprocessing

fNIRS data were preprocessed and analyzed applying the HOMER2 (version 2.2) package and customized routines in MATLAB^®^ (The MathWorks, Inc., Natick, Massachusetts, United States).

Short channel regression was performed to remove extracerebral hemodynamics to increase the sensitivity of the fNIRS measurement to deeper tissue layers, ideally to neurovascular coupling.^53^ To this end, a MATLAB^®^ function implemented by NIRx Medical Technologies was utilized based on Saager and Berger^54^ and Scholkmann et al.^55^

Optical density time series were converted to $\left[O_{2}\mathrm{Hb}\right]$, [HHb], and [tHb] time series by the modified Beer–Lambert law^56^ using HOMER2 routines (hmrIntensity2OD, hmrSSR, and hmrOD2Conc) assuming a fixed differential path-length factor value of 6.^57^ The absorption coefficients were calculated based on the extinction coefficients from Kollias and Gratzer.^58^

In our analysis, we also considered fNIRS signals without applying short channel regression ($\left[O_{2}\mathrm{Hb}\right]$ (raw)], [HHb] (raw), [tHb] (raw)) to investigate the impact of short channel regression on the results. Some fNIRS time series had to be excluded from further analysis due to an insufficient signal-to-noise ratio. The exclusion was performed based on the coefficients of variation (CV). Time series with a CV above 7.5%^59^ were excluded from the successive fNIRS analysis due to insufficient data quality. All data were screened visually for movement artifacts. No relevant artifacts were found. Based on the location of the optodes [see Fig. 1(d)], three regions of interest (ROI) were determined: the left, right, and the entire prefrontal region of the head.

The channels belonging to the same ROI were averaged. As a result, 18 fNIRS signals were obtained: $\left[O_{2}\mathrm{Hb}\right]$, [HHb], and [tHb] (i) of the left prefrontal region ($\left[O_{2}\mathrm{Hb}\right]$ (left), [HHb] (left), [tHb] (left), $\left[O_{2}\mathrm{Hb}\right]$ (left, raw), [HHb] (left, raw), and [tHb] (left, raw)), (ii) of the right prefrontal region ($\left[O_{2}\mathrm{Hb}\right]$ (right), [HHb] (right), [tHb] (right), $\left[O_{2}\mathrm{Hb}\right]$ (right, raw), [HHb] (right, raw), and [tHb] (right, raw)), (iii) and all channels ($\left[O_{2}\mathrm{Hb}\right]$ (left + right), [HHb] (left + right), [tHb] (left + right), $\left[O_{2}\mathrm{Hb}\right]$ (left + right, raw), [HHb] (left + right, raw), and [tHb] (left + right, raw)). The fNIRS time series of each experiment were then synchronized to the systemic physiological time series.

### 2.4 Data Analysis

**Fig. 2:**
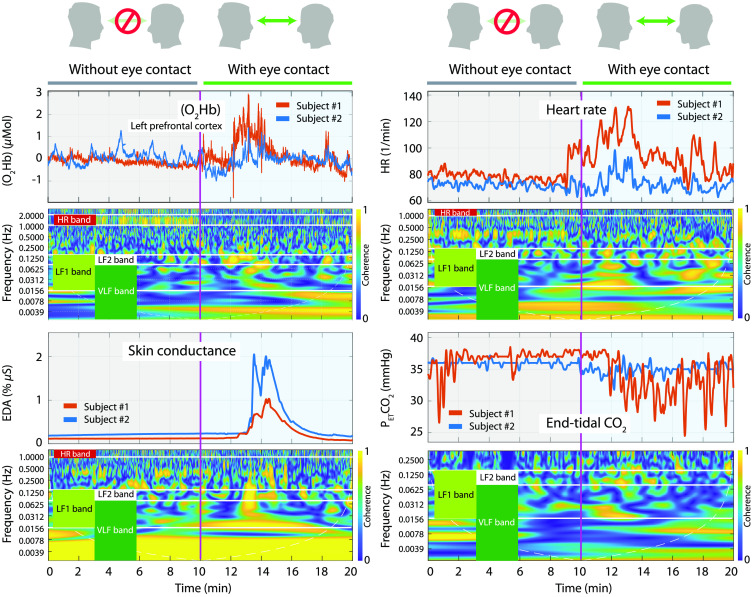
Illustrative example of physiological and fNIRS time series of one pair and the respective wavelet coherence. The figure shows the four frequency bands that have been taken into account in the analysis.

The time- and frequency-dependent correlation between the time series was determined for each type of signal and pair of participants using the wavelet transform coherence (WTC) analysis.^60^ Figure 2 provides an example of fNIRS and systemic physiological signals with the corresponding WTC analysis results. For each pair of time series, we considered the mean of the coherence calculated for four frequency bands: very low-frequency band (VLF, 0.002 to 0.08 Hz), low-frequency band 1 (LF1, 0.015 to 0.15 Hz), low-frequency band 2 (LF2, 0.08 to 0.15 Hz), and heart rate band (HR, 1 to 2 Hz). Thus, four WTC time series for each of the 29 pairs of times series were obtained.

**Fig. 3:**
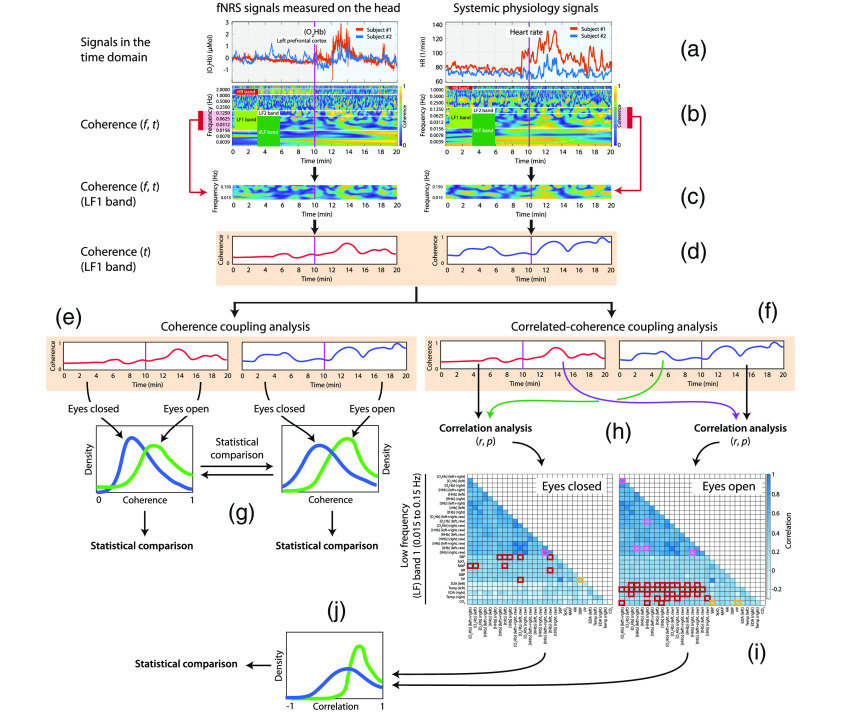
Visualization of the signal processing and data analysis steps of the coherence and correlated-coherence coupling analysis. The figure shows the steps performed in the analysis for a given dyad taking as an example, a pair of fNIRS signals ($\left[O_{2}\mathrm{Hb}\right]$ (left)) and the HR of the two participants (a) considering only the LF1 band. (b) The wavelet coherence analysis of the two pairs of time series is performed and the respective scalograms are shown with the four frequency bands. (c) One frequency band is selected and (d) the median of the coherence values is calculated, leading to two time-dependent WTC signals. (e) In the coherence coupling analysis, the time-dependent coherence of the fNIRS and systemic physiology signals have been averaged over the two conditions. (g) The ART ANOVA was then performed on the resulting coherence values to assess any difference between the eyes-closed and eye-contact conditions. (f) In the correlated-coherence coupling analysis, the time-dependent coherence signals are split according to the two conditions and (h) Spearman correlation and its significance levels are calculated, (i) leading to a correlation matrix of the correlation coefficients from all types of time series. (j) Finally, the correlation distribution of each type of time series over the two conditions was tested with Kolmogorov–Smirnov test.

For each dyad, the WTC between the same pair of time series was calculated [Figs. 3(a) and 3(b)]. Then, for each of the four frequency bands [Fig. 3(c)], the median of the coherence values was calculated, leading to one time-dependent WTC signal [Fig. 3(d)] for each pair of time series. This resulted in (29×4)−1 time-dependent WTC signals for each dyad, where the −1 represents the absence of the $P_{\mathrm{ET}}{\mathrm{CO}}_{2}$ time series in the HR frequency band as illustrated in the bottom right plot in Fig. 2. The coherence values outside the cone of influence were excluded from the further WTC analyses.

In our analysis, we only investigated the coupling between subjects, not within a subject, since the focus of our study was to investigate the coupling between subjects, i.e., the brain-to-brain, brain-to-body, and body-to-body coupling.

#### 2.4.1 Coherence coupling analysis

The time-dependent coherence of the fNIRS and systemic physiology time series was averaged over the two conditions. Thus, for each frequency band we obtained two coherence values $c_{SnEC}$ and $c_{SnEO}$, where *Sn* is one of the 29 possible WTC signals and indexes EC and EO indicate the eyes-closed and eyes-open conditions, respectively [Fig. 3(e)]. The aligned rank transformation analysis of variance (ART ANOVA),^61^ a nonparametric ANOVA, was performed to assess any difference between the two conditions [Fig. 3(g)]. In addition to the condition, also the group factor, consisting of two levels (familiar and unfamiliar pair) and the respective interaction (condition × group) were evaluated. Finally, a false discovery rate (FDR) correction was applied to the p-values to correct the multiple comparison situation. This analysis was conducted in R.

As a control analysis, we calculated the WTC for each type of signal for 2400 random pairs with replacement. The WTC values obtained by this procedure were then averaged over the two conditions and the four frequency bands. For each type of signal and each frequency band, we compared the distribution of true WTC values with that obtained from the randomly shuffled pairs by performing the Wilcoxon rank-sum test. This analysis was performed to assess that the functional hyperconnectivity between pairs occurred because of social interaction.

#### 2.4.2 Correlated-coherence coupling analysis

The correlation between each time-dependent WTC signal and the remaining 28 time-dependent WTC signals was evaluated [Fig. 3(h)]. The Spearman correlation (r) and the significance level (p-value) were calculated, generating a 28×28 matrix of WTC time-series correlation coefficients for each individual pair, each condition (eyes-closed and eye contact) and each frequency band [Fig. 3(i)].

**Fig. 4:**
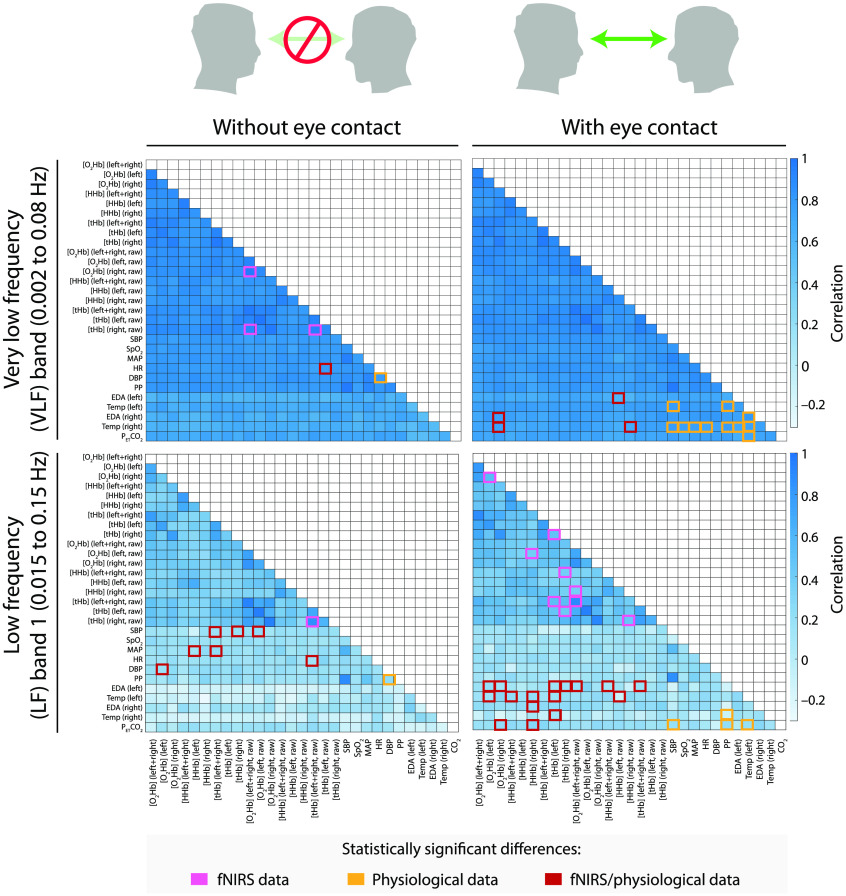
Group averages of the correlation matrices comparing the eyes-closed and eyes-open conditions in the VLF band (top left and top right, respectively) and LF1 band (bottom left and bottom right, respectively). In the two frequency bands, a marked matrix value indicates a significant difference and a higher median of the distribution than the other condition of the respective frequency band.

**Fig. 5:**
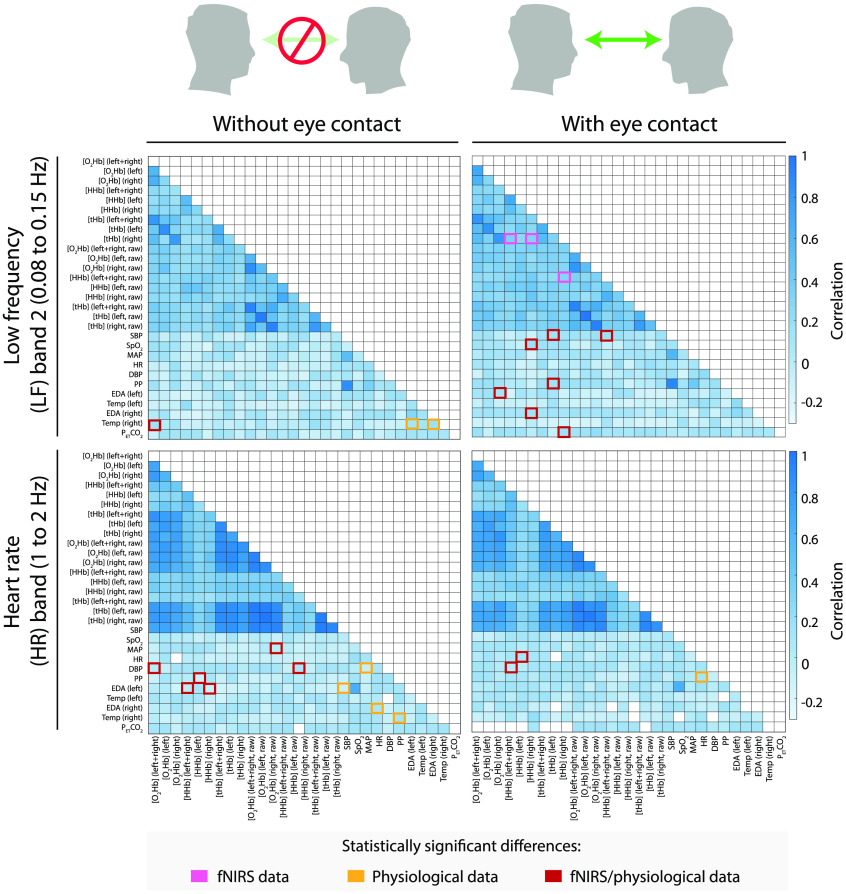
Group averages of the correlation matrices comparing the eyes-closed and eyes-open conditions in the LF2 band (top left and top right, respectively) and HR frequency band (bottom left and bottom right, respectively). In the two frequency bands, a marked matrix value index indicates a significant difference and a higher median of the distribution than the other condition of the respective frequency band.

Then, a group average was performed by calculating the median of all the individual correlation matrices, obtaining a single correlation matrix for each condition and frequency band, as illustrated in Figs. 4 and 5. For the group average, only correlation coefficients were used that were statistically significantly different from 0 (p<0.05) to ensure that the group average was based only on meaningful physiological changes from the individual measurements.

**Fig. 6:**
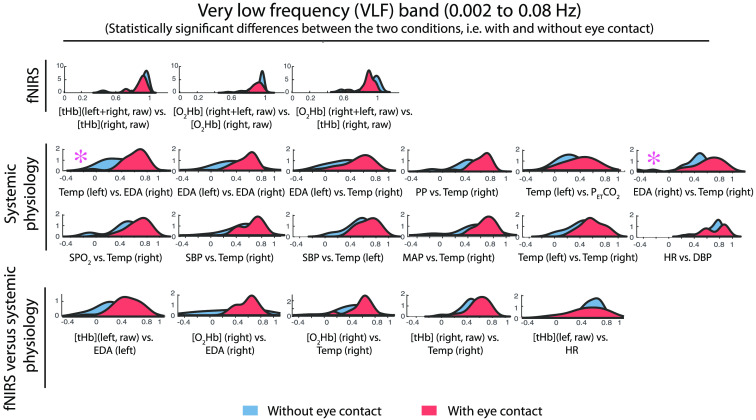
Probability density functions of the distributions of each correlation coefficient of the 24 pairs in the VLF band separated into three groups: fNIRS, physiological, and fNIRS-physiological data. The probability density functions relative to the eye-contact condition are shown in red and those relative to the eyes-closed condition in blue. In the above figure are reported only the significant differences as a result of the Kolmogorov–Smirnov test. An asterisk indicates that the p-values are significant after the FDR correction.

**Fig. 7:**
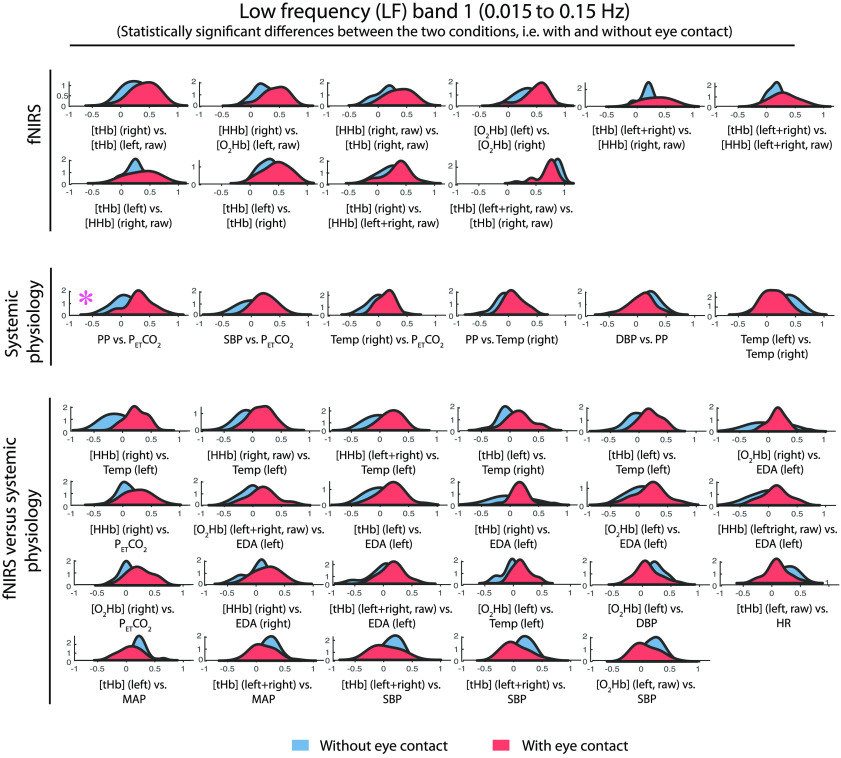
Probability density functions of the distributions of each correlation coefficient of the 24 pairs in the LF1 band separated into three groups: fNIRS, physiological, and fNIRS-physiological data. The probability density functions relative to the eye-contact condition are shown in red and those relative to the eyes-closed condition in blue. In the above figure are reported only the significant differences as a result of the Kolmogorov–Smirnov test. An asterisk indicates that the p-values are significant after the FDR correction.

**Fig. 8:**
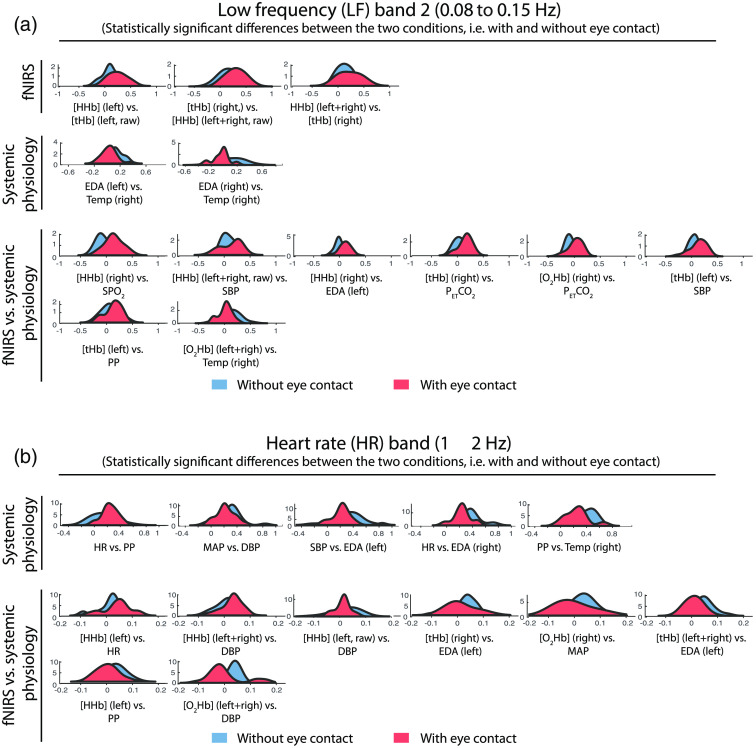
Probability density functions of the distributions of each correlation coefficient of the 24 pairs in (a) the LF1 and (b) HR band separated into three groups: fNIRS, physiological, and fNIRS-physiological data. The probability density functions relative to the eye-contact condition are shown in red and those relative to the eyes-closed condition in blue. In the above figure are reported only the significant differences as a result of the Kolmogorov–Smirnov test.

To investigate whether the correlation distribution of each pair of time series during the eye-contact condition was statistically different from the one during the eyes-closed condition, in each frequency range the Kolmogorov–Smirnov test was performed [Fig. 3(j)]. The significant results are visualized in Figs. 6–8 separated into four bands (VLF, LF1, LF2, and HR) and three groups: fNIRS, systemic physiological and fNIRS/systemic physiological data.

In addition, the difference in the correlation distribution between familiar pairs and unfamiliar pairs groups was assessed. First, the difference of the correlation matrix values evaluated during the eye-contact condition and that during the eyes-closed condition for each of the four bands was computed. Next, a group average was performed by determining the median split for the familiar pairs and unfamiliar pairs as depicted in Figs. S1 and S2 in the Supplemental Materials. The difference between the distributions of the two groups was finally evaluated by Kolmogorov–Smirnov test in each frequency range.

## 3 Results

### 3.1 Coherence Coupling Analysis

#### 3.1.1 Differences between eyes closed and open

The fNIRS results of the ART ANOVA are summarized in Table S1 in the Supplemental Materials, and the systemic physiology results are summarized in Table S2 in the Supplemental Materials.

**Fig. 9:**
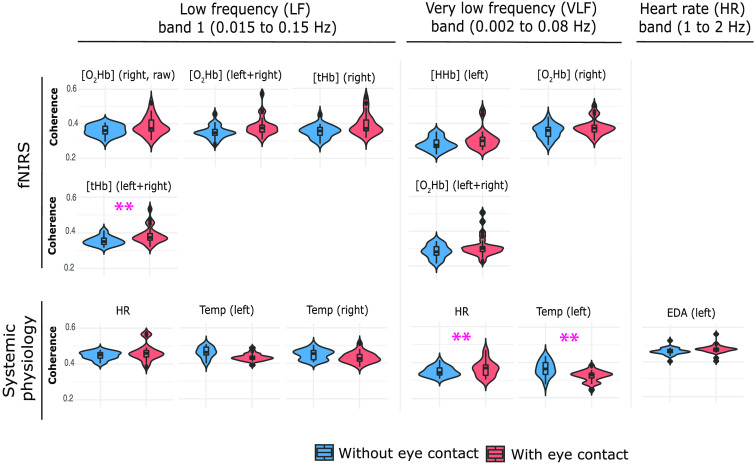
Violin plots of the distributions of the WTC values over the two conditions separated into three frequency bands (LF1, VLF, and HR) and two groups: fNIRS and systemic physiology signals. In the above figure are reported only the significant differences as a result of the ART ANOVA. Double magenta asterisks refer to statistically significant differences between the two conditions even after FDR correction.

Significant results related to the difference between the two conditions are illustrated in Fig. 9.

After performing an FDR correction, results showed a significant difference between the two conditions in [tHb] (left + right) (LF1 band, p=0.001) (Table S1 in the Supplemental Materials), in Temp (left) and HR (VLF band, p=0.010 and p=0.003, respectively) (Table S2 in the Supplemental Materials). While both [tHb] (left + right) and HR showed a higher coherence in eye-contact condition compared with eyes-closed condition, Temp (left) presented an opposite effect (eye-contact coherence < eyes-closed coherence) (Fig. 9).

Additional significant coherence occurred without FDR correction where coherence during eye contact appeared higher in fNIRS signals in the VLF band, $\left[O_{2}\mathrm{Hb}\right]$ (left) (p=0.012), $\left[O_{2}\mathrm{Hb}\right]$ (right) (p=0.043), $\left[O_{2}\mathrm{Hb}\right]$ (left + right) (p=0.031), and in the LF1 band, $\left[O_{2}\mathrm{Hb}\right]$ (right, raw) (p=0.017), $\left[O_{2}\mathrm{Hb}\right]$ (left + right) (p=0.016), [tHb] (right) (p=0.004) as summarized in Table S1 in the Supplemental Materials and illustrated in Fig. 9. No difference was observed between the corrected fNIRS signals and those without applying the short channel regression.

Regarding the systemic physiology signals, EDA (left), in the HR band, and HR, in the LF1 band, showed a statistically higher coherence in the eye-contact condition compared with the other one (p=0.004 and p=0.011, respectively), while pairs exhibited a higher correlation in Temp (left) and Temp (right) signals when they were not looking at each other (LF1 band, p=0.010 and p=0.041, respectively) as listed in Table S2 in the Supplemental Materials and depicted in Fig. 9.

**Fig. 10:**
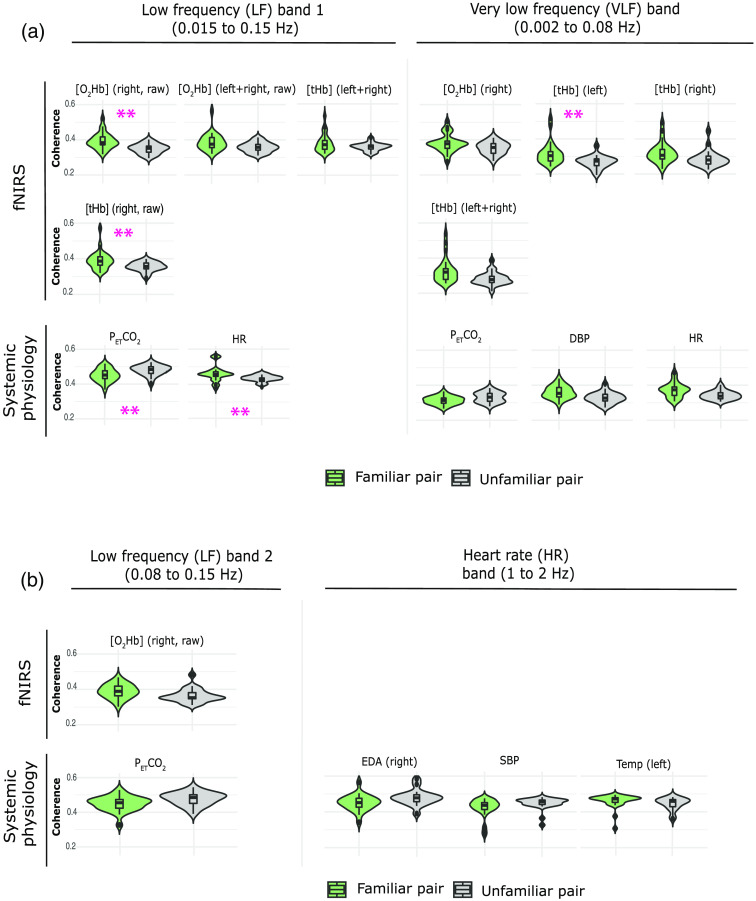
Violin plots of the WTC values distributions over the two groups (familiar and unfamiliar pair) grouped into fNIRS and systemic physiology signals and separated into (a) LF1 and VLF bands and (b) LF2 and HR bands. In the above figures are reported only the significant differences as a result of the aligned ranks transformation ANOVA. Double magenta asterisks refer to statistically significant differences between the two conditions even after FDR correction.

**Fig. 11:**
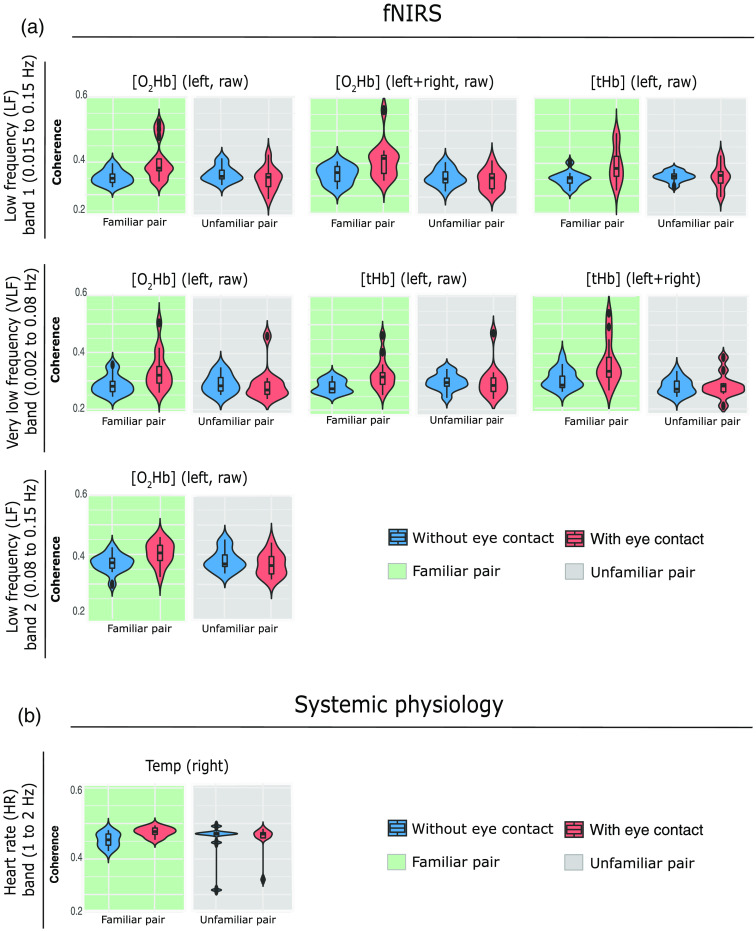
Violin plots of the WTC values distributions over the two groups (familiar and unfamiliar pair) grouped into fNIRS and systemic physiology signals and separated into (a) LF1 and VLF bands and (b) LF2 and HR bands. In the above figures are reported only the significant differences as a result of the aligned ranks transformation ANOVA.

It is important to note that the outliers in the violin plots shown in Figs. 9–11 do not impact the results since ART ANOVA is based on ranks and therefore not affected by extreme values.

#### 3.1.2 Impact of the type of relationship

The pairs who took part in our study belonged to two different groups according to their type of relationship: the first group included siblings and couples (spouses and boy/girlfriends) and the second pairs did not know each other or to a lesser extent than the other group.

After performing the FDR correction, our results showed that the coupling in the familiar pairs was higher than in the other group in the LF1 band for $\left[O_{2}\mathrm{Hb}\right]$ (right, raw) (p<0.001), [tHb] (right, raw) (p=0.004), HR (p=0.004), and in VLF band for [tHb] (left) (p<0.001). On the contrary, in the case of $P_{\mathrm{ET}}{\mathrm{CO}}_{2}$ in the LF1 band, the correlation was found to be higher in unfamiliar pairs (p=0.004). These results are reported in Tables S1 and S2 in the Supplemental Materials and are visualized in Fig. 10.

Without FDR correction, other significant differences in the coherence were noted. In all significant fNIRS results, the familiar pairs exhibited a higher hyperconnectivity. These fNIRS signals are $\left[O_{2}\mathrm{Hb}\right]$ (right), [tHb] (right), and [tHb] (left + right) in the VLF band (p=0.014, p=0.021, and p=0.006, respectively), $\left[O_{2}\mathrm{Hb}\right]$ (left + right, raw) and [tHb] (left + right) in the LF1 band (p=0.016 and p=0.014, respectively), and $\left[O_{2}\mathrm{Hb}\right]$ (right, raw) in the LF2 band (p=0.006). The same effect (coherence familiar pairs > coherence unfamiliar pairs) was evident in DBP and HR (VLF band, p=0.014 and p=0.008, respectively) and in Temp (left) (HR band, p=0.015). The synchrony between the unfamiliar pairs was higher than the other one for $P_{\mathrm{ET}}{\mathrm{CO}}_{2}$ (LF2 band, p=0.019; VLF band, p=0.022), EDA (right) (HR band, p=0.045), and SBP (HR band, p=0.026).

#### 3.1.3 Relation between the experimental conditions and the two groups

The statistically significant results without applying the FDR correction considering the two factors interaction (condition and group) is visualized in Fig. 11. In all the signals, the familiar pair group always shows a higher coherence during the eye contact compared with the eyes-closed condition coherence. In addition, the difference between eye-contact and eyes-closed conditions in the familiar pairs is statistically higher than the difference between the eye-contact and eyes-closed conditions in the unfamiliar pairs. No significant effect was found after performing the FDR correction. The posthoc statistics of the significant results are available in Table S3 in the Supplemental Materials.

#### 3.1.4 Control analysis

The fNIRS results of the control analysis are summarized in Table S4 in the Supplemental Materials, while the systemic physiology results are listed in Table S5 in the Supplemental Materials. After performing the FDR correction, our results revealed that the coherence in the real pairs were statistically higher than the spurious coherence during the eye-contact condition in many fNIRS signals: [HHb] (left), $\left[O_{2}\mathrm{Hb}\right]$ (right), $\left[O_{2}\mathrm{Hb}\right]$ (left + right), [tHb] (right), and [tHb] (left + right) in the LF1, LF2, and VLF band (p<0.001); [HHb] (left, raw) in the LF1 band (p<0.001), in the LF2 band (p=0.016), in the VLF band (p=0.013); [HHb] (right) in LF1 and VLF (p=0.001); [HHb] (left + right) in LF1 and VLF (p<0.001), in LF2 (p=0.009); [HHb] (left + right, raw) in VLF (p<0.001); $\left[O_{2}\mathrm{Hb}\right]$ (left) in LF1 and VLF (p<0.001), in LF2 (p=0.013); [tHb] (left) in LF1 (p<0.001), in LF2 (p=0.001) and in VLF (p=0.002). The same effect (coherence in the real pairs > spurious coherence) was found during the eyes-closed condition: [HHb] (left) and [HHb] (left + right) in LF1 band (p<0.001); [HHb] (left + right) in (p=0.015); $\left[O_{2}\mathrm{Hb}\right]$ (left + right) in LF2 band (p=0.004). The results confirm that coherence values in real pairs are statistically higher than those of random pairs in many fNIRS signals.

Regarding systemic physiology signals, there was a significant effect (real coherence > spurious coherence) in HR and EDA (left and right) but only before performing FDR correction.

### 3.2 Correlated-Coherence Coupling Analysis

#### 3.2.1 Correlations between fNIRS and systemic physiological signals

The median correlation matrices relative to the eyes-closed and eyes-open conditions are shown in Fig. 4 (for the VLF and LF1 frequency bands) and Fig. 5 (for the LF2 and HR frequency bands). From the figures it is obvious that a strong correlation exists between all signals in the VLF band, and a higher correlation between the fNIRS signals in the other three frequency bands compared with the systemic physiology variables and fNIRS-systemic physiology variables.

The significant results obtained after performing the Kolmogorov–Smirnov test are grouped into three categories (fNIRS, systemic physiology and fNIRS/systemic physiology) and are illustrated in Fig. 6 (VLF band), Fig. 7 (LF1 band), and Fig. 8 (LF2 and HR bands). Statistically significant results after performing an FDR correction were marked with an asterisk.

The probability density functions of the distributions relative to the eye-contact condition are shown in red and those relative to the eyes-closed condition in blue.

The analysis framework enables us to investigate the simultaneous correlation of two time-dependent coherence vectors [${\mathrm{WTCS}}_{1}(t)$ and ${\mathrm{WTCS}}_{2}(t)$, where $S_{1}$ and $S_{2}$ represent two biosignals] between two individuals in each frequency band. A high correlation between the two coherence vectors can be indicated either as a positive time correlation between $S_{1}$ and $S_{2}$ time series or as a negative one. This method provides a synchrony indication between two pairs of signals. The correlated-coherence coupling analysis was conducted in MATLAB^®^.

The correlation between fNIRS and systemic physiological time series was also assessed with respect to the familiar and familiar pairs. The median correlation matrices relative to the pair groups are shown in Fig. S1 (for the VLF and LF1 frequency bands) and Fig. S2 (for the LF2 and HR frequency bands) in the Supplemental Materials. No statistically significant difference was found between the two groups after performing FDR correction.

## 4 Discussion

The main goal of this study was to explore the possibility of extending the conventional fNIRS hyperscanning approach by introducing the new SPA-fNIRS hyperscanning method enabling to simultaneously assess the brain and body synchrony from two individuals during a task involving subjects that sit in front of each other while phases of having the eyes closed and open. To investigate this, we measured changes in systemic physiology and fNIRS signals while they kept their eyes closed for 10 min and while they maintained eye contact for 10 min. We intentionally used the eyes-closed condition to ensure that the visual communication channel was totally blocked, while the prolonged eye contact was explicitly chosen to capture a strong visual interaction flow and enhance the effect of the visual channel. We also aimed to investigate the temporal relationship between the brain and body by determining the coherence between physiological, fNIRS and fNIRS/physiological signals, and we examined the role played by the visual communication channel. Previous studies have evaluated such relationships between brain activity and systemic parameters on the intrapersonal level (single brain recording),^62^^–^^66^ but we are the first that also evaluated the interpersonal level. The LF1 band (i.e. 0.015–0.15 Hz) has been identified as the frequency band in which the visual channel evokes the strongest correlation between fNIRS and physiological data signals, while the VLF band (i.e. 0.002–0.08 Hz) exhibited the highest correlation between physiological signals, as shown in Fig. 6.

Our study is the first published SPA-fNIRS hyperscanning study and the first that explores the role of eye contact for functional connectivity between the brain and the body of two individuals.

Our study shows that making eye contact for a prolonged time causes significant changes in brain-to-brain, brain-to-body, and body-to-body coupling, indicating that eye contact is followed by entrainment of the physiology between subjects. Subjects that knew each other generally showed a larger trend to change between the two conditions.

### 4.1 Coherence Coupling Analysis

#### 4.1.1 Differences between eyes closed and open

Several studies have explored the importance of visual contact in social cognition so far, e.g., the importance of social attention.^67^^–^^69^ Within this context, a number of studies were performed using static images or schematic stimuli.^70^^–^^72^ Recent studies focused on face-to-face gaze rather than traditional stimulations by photo/video display.

Our study results show that pairs exhibit statistically higher coherence in physiological and fNIRS signals during face-to-face eye gaze than when their eyes were closed. Although external factors such as light coming into participants’ eyes during the eye-contact phase might cause an effect on the brain and body activity, coupling between the subjects occurred at different times when they made eye contact (as also shown in Fig. 2). Therefore, we believe that the main effect is due to the visual interaction of the subjects. A strong hyperconnectivity in [tHb] in the LF1 band of the whole prefrontal cortex was found. Since for this signal the coherence in true pairs (during the eye contact) was found to be statistically higher than the coherence in random pairs, we can conclude that it is associated with social interaction. Moreover, interbody synchrony of the HR during eye contact was observed. In the VLF band, a significant and strong coherence was observed during visual communication in the skin temperature of the left wrist and the HR. In line with our results, such synchronization in HR has already been found between mother and child during face-to-face interaction.^73^ However, since interbody synchrony in true pairs is not statistically higher than the random pairs (but only before performing the FDR correction), we cannot conclude that this effect is caused by eye contact.

Furthermore, a significant coherence during the phase of eye contact between the subjects was found in the skin temperature from both the wrists (in the LF1 band) and in EDA measured on the left wrist (in the HR band) that was not significant after the FDR correction. Concerning EDA, a similar result has been found by Jarick and Bencic^74^ where dyads showed an increase in skin conductance while making eye contact compared with nondirect eye contact. To the best of our knowledge, skin temperature is a parameter has never been investigated in a hyperscanning study before.

In addition, pairwise synchrony in the eye-contact condition compared with the eyes-closed condition was also significantly greater but only without FDR correction in $\left[O_{2}\mathrm{Hb}\right]$ throughout the whole prefrontal cortex (in the VLF band) and in [tHb] in the right prefrontal cortex (in the LF1 band). In line with our results, other studies involving the brain activity of a single subject demonstrated a difference between resting-state brain activity and brain activity when the subject was observing.^75^^–^^89^

A few fNIRS hyperscanning studies were conducted so far that investigated the difference in functional hyperconnectivity between the eyes open and eyes closed condition. Osaka et al.^90^ demonstrated a higher brain-to-brain coherence during a cooperative humming task during eye contact compare to no eye contact. Hirsch et al.^91^ found in the left frontal region a higher hyperconnectivity during the eye-to-eye contact compared with the eye-to-picture gaze condition.

Consistently with previous studies, the results of our study indicate that visual contact is a salient component and plays a key role in social interaction during which high hyperconnectivity was observed.

#### 4.1.2 Relation between the experimental conditions and the two groups

The familiar group exhibited a stronger body-to-body coupling with regard to HR (in the LF1 band) and brain-to-brain coupling in terms of [tHb] of the right prefrontal region (in the VLF band) and the raw $\left[O_{2}\mathrm{Hb}\right]$ of the right prefrontal region (in the LF2 band) compared with the unfamiliar pairs. On the other hand, the unfamiliar pairs showed a stronger body-to-body coupling in terms of $P_{\mathrm{ET}}{\mathrm{CO}}_{2}$ (in the LF1 band) compared with the familiar pairs. These findings are unique.

A higher body-to-body coupling trend in the familiar group also emerged between systemic physiology variables such as EDA and skin temperature but only without FDR correction.

Slovák et al.^92^ explored the connection between EDA and emotional engagement during a conversation task. According to their findings, the degree of synchrony between pairs depends on the strength of the empathic connection between the subjects.

The familiar group exhibited a stronger synchrony trend during eye contact than the other group in terms of skin temperature (in the HR band), $\left[O_{2}\mathrm{Hb}\right]$, and [tHb]. The lack of a statistical significance may be due to the small sample size and large variability within each group.

### 4.2 Correlated-Coherence Coupling Analysis

#### 4.2.1 Higher coherence during eye contact between physiological data

We found a significantly higher coherence between skin temperature (from both wrists) and EDA in the VLF band when the subjects had eye contact compared to the period when they had their eyes closed even after the FDR correction. EDA changes depend on the quantity of sweat secreted by eccrine sweat glands controlled by the sympathetic nervous system (SNS). If the sympathetic branch of the autonomic nervous system (ANS) is highly activated, the sweat glands activity increases, leading to higher skin conductance, and vice versa. Previous studies have shown that eye contact leads to significantly higher skin conductance compared with nonmutual gaze.^93^^–^^97^ The relationship between EDA, skin temperature, and mutual gaze has never before been investigated. Khan et al.^98^ reported results similar to ours, noting a significant positive correlation between these two variables when students performed an engineering exam. The higher correlation of two pairs EDA time series during eye contact could be attributable to an increased emotional response either as a result of affection or due to a sense of unease. This increased the EDA and the skin conductance. On the other hand, this could trigger cutaneous vasoconstriction, which results in decreased skin blood flow inducing a drop in skin temperature. In fact, skin temperature is directly correlated with peripheral blood flow.^99^ Based on this, recent studies have shown that direct eye contact between two people causes a stress reaction, which manifests itself as an increased SNS activity.^100^ As a result, the high correlation during the eyes open condition between EDA and Temp coherence vectors may be attributable to a negative time correlation between the two signals of the individual pairs.

The same pattern was observed between the skin temperature of the left wrist and $P_{\mathrm{ET}}{\mathrm{CO}}_{2}$ in the VLF band as well as between the skin temperature of the right wrist and $P_{\mathrm{ET}}{\mathrm{CO}}_{2}$ in the LF1 band. An increase in $P_{\mathrm{ET}}{\mathrm{CO}}_{2}$ coherence between pairs may theoretically arise from three sources: simultaneous decrease (or increase) in alveolar ventilation, increase (or decrease) in ${\mathrm{CO}}_{2}$ production, and the quantity of ${\mathrm{CO}}_{2}$ that contributes to the respiratory capillary circulation.^101^ Respiratory reactions induced by eye contact produce changes in ventilation that are reflected by alterations in $P_{\mathrm{ET}}{\mathrm{CO}}_{2}$. In fact, in most people, emotional arousal is linked to reduced $P_{\mathrm{ET}}{\mathrm{CO}}_{2}$ levels.^102^ This slight hypocapnia occurs when alveolar ventilation is greater than that required to eliminate metabolically formed ${\mathrm{CO}}_{2}$.^103^ Since the arousal affects both parameters simultaneously, this leads to a high coherence.

Significant coherence was found in the VLF and LF1 band between the skin temperature (of both wrists) and cardiovascular variables such as HR, PP, SBP, SpO_2_ and MAP. Such cardiovascular variations may be interpreted as an SNS response related to the pair’s interaction.^104^

In addition, a significant symmetry in both wrists between EDAs as well as skin temperatures was found in the VLF band. In the LF1 band instead, coherence in $P_{\mathrm{ET}}{\mathrm{CO}}_{2}$ was found to be correlated to the coherence in PP (also after the FDR correction) and SBP.

In fact, during eye contact, the cardiovascular variables are affected by the ANS. Both branches of the ANS (parasympathetic nervous system and SNS) are of significant importance in controlling cardiovascular dynamics, including HR, MAP regulation, and respiration,^105^^,^^106^ but they have contrary effects on the functions they regulate. In case of possible discomfort, due to the mutual gaze of the pairs, the SNS is more activated, causing an increase in MAP and, at the same time, in skin conductance.

Several studies have investigated and found an asymmetry between the right and left sides of the human body related to the measurement of skin temperature and EDA.^107^^–^^110^ These results could explain the reason why some variables in our study are correlated only with EDA and/or skin temperatures on the right wrist and not on the left and vice versa. Besides, this relationship was found to be stronger on the right wrist in accordance with Demirel et al.^107^ According to Demirel et al., such asymmetry may be associated with the peripheral immune asymmetry or asymmetric lymph node distribution.

In our results, skin temperature plays a predominant role. In fact, correlations with several cardiovascular and EDA parameters have been found, and most importantly, the correlation during eye contact has been found to be more significant than that period when the subject had their eyes closed.

#### 4.2.2 Higher coherence during eye contact between physiological and fNIRS data

Several significant temporal relationships between fNIRS and systemic physiological signals were identified, mainly in the LF1 (see Fig. 7). This is an indicator that the fNIRS signal was influenced by changes in systemic physiology.^42^^,^^46^^,^^111^^,^^112^ The fNIRS signals measured therefore represent a weighted sum of physiological changes due to neurovascular coupling as well as due to different physiological causes (e.g. changes in systemic physiology).

The strong coherence in all frequency bands between fNIRS signals and EDA is the most prominent finding. This is in line with previous publications that focused on investigating a relationship between brain hemodynamics and oxygenation with EDA on the intrapersonal level.^62^^–^^65^ This relationship was found to be dependent on the type of task the participants were involved, as discussed by Holper et al.^66^

Another remarkable finding is the higher coherence in the signal phase during eye contact between skin temperature and fNIRS signals, especially in the LF1 frequency band. Concerning the skin temperature, we found an interesting pattern: While the correlation between physiological signals and skin temperature was higher in the right arm in the LF1 and VLF bands, the correlation between fNIRS signals and skin temperature in the LF1 band was predominant in the left wrist. This is a unique finding in this study.

In addition, a significant correlation between the fNIRS variable and the $P_{\mathrm{ET}}{\mathrm{CO}}_{2}$ was obtained, only for the right prefrontal region in the LF1 and LF2 bands.

Our results suggest the presence of interconnection between cerebral hemodynamic variables and SNS activity. Although such relationships have been reported previously, we are the first to identify them in investigating the long-term effects of eye contact in the context of SPA-hyperscanning.

#### 4.2.3 Higher coherence during eye contact between fNIRS data

The strongest correlation during eye contact of fNIRS occurred in the LF1 and LF2 bands. No evident pattern has arisen apart from a greater symmetry between the left and right region in $\left[O_{2}\mathrm{Hb}\right]$ as well as in [tHb] in the LF1 band during the eye contact period compared with the period with eyes closed.

#### 4.2.4 Higher coherence during eyes-closed condition

A lower coherence was observed in some signals in all four frequency bands during the eyes’ contact than when eye contact was blocked. We have found no explanation for this phenomenon.

## 5 Strengths and Limitations

We are the first group to perform a SPA-fNIRS hyperscanning study capturing changes in cerebral oxygenation and hemodynamics as well as systemic physiology in two subjects in parallel. We performed an advanced signal analysis to capture the coupling of the signals in the time-frequency domain within and between the two subjects, and we had enough subjects to perform a subgroup analysis enabling us to find differences in the coupling between pairs of subjects according to their relationship.

Our experimental study allowed us to test the feasibility of our novel framework and to prove that, with a simple experimental design, it is already possible to identify significant changes in biosignals that have not been investigated in detail in previous studies (using fNIRS or fMRI). There are many possible extensions to our data analysis. For example, cross-frequency coupling could also be examined in the future.

Although our primary focus was to assess the influence of the visual communication channel, an interesting step in the further investigation might be to consider a third condition, in which participants keep their eyes open without making eye contact, to exclude that part of the effect might be due only by the difference between eyes-open versus eyes-closed conditions.

It should be pointed out that, although in everyday life there are scenarios in which prolonged eye contact plays an important role in social interaction (e.g., the conversation between friends, couples, and interviews), keeping eye contact for 10 min something that happens rather rarely in everyday life.

A limitation of our approach might be the lack of an eye-tracking system in our setup and, therefore, a precise temporal indication of when eye contact occurred and how long it lasted is missing. The lack of brain/body coupling we found for specific individuals may be associated, not only with how subjects are related but also with too short a gaze to achieve brain-to-brain and body-to-body synchrony, due to the greater discomfort perceived by one person in staring into the eyes of another person.

Within this context, automatic identification of a subset of relevant features for each pair is crucial for a robust building of a synchronization model, which considers actual eye contact. The difference between the cross-brain correlations of the two groups is attributable to the participants’ relationships. Individuals who know each other well connect with a higher empathy. This assumption might be addressed in future studies exploring brain and body synchrony of different groups such as mother-child and nurse-child.

## 6 Conclusions

The main goal of this exploratory study was to provide a new framework for assessing brain-to-brain, brain-to-body, and body-to-body coupling between interacting subject pairs that can be adopted in future hyperscanning studies. To give an example of our novel SPA-fNIRS hyperscanning approach, we tested it on a simple social experimental paradigm while enabling or disabling visual communication between the subject pairs.

We found by the coherence coupling analysis that the eye contact generally causes an increase in brain-to-brain coupling (e.g., [tHb] in the LF1 band) and characteristic changes in the body-to-body coupling (e.g., an increase of the HR in the VLF band a decrease in the temperature of the left wrist also in the VLF band). The changes due to the eye contact showed a relationship-dependent trend, i.e., pairs that were well acquainted with each other showed a higher increase in brain-to-brain coupling (in the LF and VLF bands) and an increase/decrease in body-to-body coupling (with regard to $P_{\mathrm{ET}}{\mathrm{CO}}_{2}$ and HR in the LF band, respectively) compared with the phase without eye contact. Our correlated-coherence coupling analysis revealed complex changes in the correlation of coupling changes between several brain and body-related physiological signals.

Our study shows that looking at each other for a prolonged time causes significant changes in brain-to-brain, brain-to-body, and body-to-body coupling, indicating that looking at each other is accompanied by entrainment of the physiology between subjects. This has implications for all life situations where eye-to-eye contact is happening for a prolonged time, e.g., during a teaching situation (pupils versus teacher and students versus lecturer) or a dialogue (e.g., interview, psychological consultation, or team meeting).

Our study is also the first that employed the SPA-fNIRS approach and showed its usefulness to investigate complex interpersonal physiological changes. We recommend for future fNIRS hyperscanning studies to also use the SPA-fNIRS approach since it provides much more information and insights into the physiological coupling between subjects compared with the classical fNIRS approach that only focuses on the brain. Both the brain and the body coupling need to be assessed to have a comprehensive understanding of physiological changes associated with social cognition. SPA-fNIRS has a great potential for social neuroscience to explore physiological couplings in various real-life conditions and circumstances.

## Code, Data, and Materials Availability

The data are not publicly available due to legal and ethical reasons.
