# Supplementary material for: Systemic physiology augmented functional near-infrared spectroscopy hyperscanning: a first evaluation investigating entrainment of spontaneous activity of brain and body physiology between subjects
Source: Neurophotonics. 2022 Apr 18;9(2):026601. doi: 10.1117/1.NPh.9.2.026601 (PMC9016073; doi:10.1117/1.NPh.9.2.026601)
Supplement: Supplementary file 1 [file NPh_009_026601_SD001.pdf]

Supplementary material

Table S1 Aligned-rank transform results of fNIRS coherence coupling analysis for LF1 (0.015–0.15 Hz), LF2 (0.08–0.15 Hz), VLF (0.002–0.08 Hz) and HR (1–2 Hz) frequency band. In the table are listed the degrees of freedom (Df), degrees of freedom for the residual (res), the F value for each effect (F), the partial eta-squared (ηp2), and the *p*-values for the F statistics (Pr(>F)). A single asterisk indicates $p < 0.05$, while the double asterisks indicate that the *p*-values are significant after the false discovery rate correction. All the significant results are marked in bold typeface, where a single asterisk indicates $p < 0.05$ and the double asterisks indicate that the p-values are significant after the FDR correction.

				**LF1 band**			**LF2 band**			**VLF band**			**HR band**		
**Signal**	**Term**	**Df**	**res**	***F***	η_p^2	***Pr(>F)***	***F***	η_p^2	***Pr(>F)***	***F***	η_p^2	***Pr(>F)***	***F***	η_p^2	***Pr(>F)***
**[HHb] (left)**	Condition	1	42	2.217	0.05	0.144	1.111	0.03	0.298	3.033	0.07	0.089	0.006	<0.01	0.940
	Group	1	42	0.012	<0.01	0.915	1.039	0.02	0.314	0.204	<0.01	0.654	2.911	0.06	0.095
	Cond.:Group	1	42	1.067	0.02	0.308	0.092	<0.01	0.763	0.821	0.02	0.370	0.005	<0.01	0.945
**[HHb] (left, raw)**	Condition	1	42	0.019	<0.01	0.890	0.671	0.02	0.417	1.261	0.03	0.267	0.287	0.01	0.595
	Group	1	42	2.398	0.05	0.129	1.137	0.03	0.292	<0.01	<0.01	0.984	0.794	0.02	0.378
	Cond.:Group	1	42	0.084	<0.01	0.773	0.777	0.02	0.383	0.841	0.02	0.364	0.023	<0.01	0.881
**[HHb] (right)**	Condition	1	44	0.390	0.01	0.535	1.268	0.03	0.266	0.731	0.02	0.397	0.146	<0.01	0.704
	Group	1	44	0.477	0.01	0.493	0.198	<0.01	0.659	0.058	<0.01	0.811	0.006	<0.01	0.936
	Cond.:Group	1	44	0.010	<0.01	0.921	0.467	0.01	0.498	0.340	0.01	0.563	0.043	<0.01	0.837
**[HHb] (right,raw)**	Condition	1	44	1.971	0.04	0.167	2.763	0.06	0.104	0.105	<0.01	0.748	0.003	<0.01	0.954
	Group	1	44	0.974	0.02	0.329	0.260	0.01	0.613	0.500	0.01	0.483	1.496	0.03	0.228
	Cond.:Group	1	44	1.886	0.04	0.177	0.051	<0.01	0.822	0.224	0.01	0.638	0.126	<0.01	0.724
**[HHb] (left+right)**	Condition	1	44	0.043	<0.01	0.837	0.511	0.01	0.479	2.150	0.05	0.150	<0.01	<0.01	0.989
	Group	1	44	0.002	<0.01	0.968	2.461	0.05	0.124	0.528	0.01	0.471	0.906	0.02	0.346
	Cond.:Group	1	44	0.052	<0.01	0.821	0.607	0.01	0.440	0.134	<0.01	0.716	0.100	<0.01	0.754
**[HHb] (left +right, raw)**	Condition	1	44	1.118	0.02	0.296	0.843	0.02	0.364	0.019	<0.01	0.892	0.156	<0.01	0.695
	Group	1	44	0.015	<0.01	0.904	3.969	0.08	0.053	0.684	0.02	0.413	0.040	<0.01	0.842
	Cond.:Group	1	44	1.699	0.04	0.199	0.804	0.02	0.375	0.005	<0.01	0.941	0.052	<0.01	0.821
**[O_2Hb] (left)**	Condition	1	42	1.576	0.04	0.216	0.430	0.01	0.515	6.949	0.14	**0.012***	0.117	<0.01	0.734
	Group	1	42	0.079	<0.01	0.780	0.908	0.02	0.346	3.112	0.07	0.085	<0.01	<0.01	0.983
	Cond.:Group	1	42	0.003	<0.01	0.956	0.014	<0.01	0.908	0.024	<0.01	0.878	0.006	<0.01	0.937
**[O_2Hb] (left, raw)**	Condition	1	42	0.867	0.02	0.357	1.109	0.02	0.298	2.098	0.05	0.155	0.262	0.01	0.611
	Group	1	42	1.294	0.03	0.261	0.178	<0.01	0.675	2.464	0.05	0.124	0.006	<0.01	0.936
	Cond.:Group	1	42	10.08	0.19	**0.003***	4.285	0.09	**0.044***	6.114	0.12	**0.017***	0.188	<0.01	0.667
**[O_2Hb] (right)**	Condition	1	44	1.715	0.04	0.197	1.141	0.03	0.291	4.341	0.09	**0.043***	0.382	0.01	0.540
	Group	1	44	3.219	0.07	0.080	0.961	0.02	0.332	6.609	0.13	**0.014***	0.040	<0.01	0.842
	Cond.:Group	1	44	0.219	<0.01	0.642	3.177	0.07	0.082	0.012	<0.01	0.913	0.002	<0.01	0.963
**[O_2Hb] (right, raw)**	Condition	1	44	6.112	0.12	**0.017***	3.181	0.07	0.081	3.043	0.06	0.088	0.176	<0.01	0.677
	Group	1	44	18.33	0.29	**<0.001****	8.165	0.16	**0.006***	1.259	0.03	0.268	0.090	<0.01	0.765
	Cond.:Group	1	44	3.382	0.07	0.073	0.138	<0.01	0.712	0.006	<0.01	0.939	0.069	<0.01	0.794

$[O_2Hb]$ (left + right)	Condition	1	44	6.258	0.12	**0.016***	0.691	0.02	0.410	4.940	0.10	**0.031***	0.462	0.01	0.500
	Group	1	44	2.843	0.06	0.099	0.040	<0.01	0.842	3.961	0.08	0.053	0.006	<0.01	0.936
	Cond.:Group	1	44	0.102	<0.01	0.751	1.118	0.02	0.296	0.098	<0.01	0.756	0.055	<0.01	0.816
$[O_2Hb]$ (left + right, raw)	Condition	1	44	2.060	0.04	0.158	0.416	0.01	0.522	1.957	0.04	0.169	0.246	0.01	0.623
	Group	1	44	6.308	0.13	**0.016***	0.033	<0.01	0.858	3.735	0.08	0.060	0.389	0.01	0.536
	Cond.:Group	1	44	5.084	0.10	**0.029***	0.098	<0.01	0.756	1.138	0.03	0.292	0.026	<0.01	0.873
[tHb] (left)	Condition	1	42	0.662	0.02	0.420	1.854	0.04	0.181	3.792	0.08	0.058	0.426	0.01	0.518
	Group	1	42	3.025	0.07	0.089	0.012	<0.01	0.915	20.584	0.33	**<0.001****	0.090	<0.01	0.766
	Cond.:Group	1	42	0.665	0.02	0.420	1.180	0.03	0.284	2.150	0.05	0.150	0.001	<0.01	0.977
[tHb] (left, raw)	Condition	1	44	3.406	0.07	0.072	2.161	0.05	0.149	2.339	0.05	0.133	0.188	<0.01	0.667
	Group	1	44	0.530	0.01	0.471	0.006	<0.01	0.937	1.341	0.03	0.253	0.002	<0.01	0.968
	Cond.:Group	1	44	4.820	0.10	**0.033***	3.265	0.07	0.078	5.283	0.11	**0.026***	0.189	<0.01	0.666
[tHb] (right)	Condition	1	44	8.978	0.17	**0.004***	5.405	0.11	**0.025***	1.000	0.02	0.323	0.317	0.01	0.576
	Group	1	44	3.085	0.07	0.086	0.317	0.01	0.576	5.688	0.11	**0.021***	0.004	<0.01	0.952
	Cond.:Group	1	44	0.136	<0.01	0.714	2.099	0.05	0.154	0.857	0.02	0.360	0.009	<0.01	0.925
[tHb] (right,raw)	Condition	1	44	1.927	0.04	0.172	2.758	0.06	0.104	1.042	0.02	0.313	0.177	<0.01	0.676
	Group	1	44	9.278	0.17	**0.004****	3.602	0.08	0.064	1.402	0.03	0.243	0.213	<0.01	0.647
	Cond.:Group	1	44	3.009	0.06	0.090	0.309	0.01	0.581	0.611	0.01	0.438	0.018	<0.01	0.894
[tHb] (left + right)	Condition	1	44	12.348	0.22	**0.001****	2.923	0.06	0.094	1.649	0.04	0.206	0.252	0.01	0.618
	Group	1	44	6.575	0.13	**0.014***	1.113	0.02	0.297	8.525	0.16	**0.006***	0.006	<0.01	0.937
	Cond.:Group	1	44	2.873	0.06	0.097	0.528	0.01	0.471	4.593	0.09	**0.038***	0.064	<0.01	0.801
[tHb] (left + right, raw)	Condition	1	44	2.645	0.06	0.111	0.135	<0.01	0.715	0.786	0.02	0.380	0.497	0.01	0.485
	Group	1	44	2.680	0.06	0.109	0.587	0.01	0.448	<0.01	<0.01	0.984	0.273	0.01	0.604
	Cond.:Group	1	44	4.270	0.09	0.045	0.755	0.02	0.390	0.195	<0.01	0.661	0.010	<0.01	0.919

**$p < 0.05$; ** significant p-values after the FDR correction.*

Table S2 Aligned-rank transform results of systemic physiology coherence coupling analysis.

Signal	Term	Df	res	LF1 band			LF2 band			VLF band			HR band		
				F	η_p^2	*Pr*(>*F*)	*F*	η_p^2	*Pr*(>*F*)	*F*	η_p^2	*Pr*(>*F*)	*F*	η_p^2	*Pr*(>*F*)
**$P_{ET}CO_2$**	Condition	1	42	1.652	0.04	0.206	1.514	0.03	0.225	2.765	0.06	0.104	*NA*	*NA*	*NA*
	Group	1	42	9.510	0.18	**0.004****	5.914	0.12	**0.019***	5.656	0.12	**0.022***	*NA*	*NA*	*NA*
	Cond.:Group	1	42	0.811	0.02	0.373	2.695	0.06	0.108	2.555	0.06	0.117	*NA*	*NA*	*NA*
**DBP**	Condition	1	42	0.006	<0.01	0.938	0.193	<0.01	0.663	0.199	<0.01	0.658	2.325	0.05	0.135
	Group	1	42	0.077	<0.01	0.783	3.725	0.08	0.060	6.555	0.14	**0.014***	2.414	0.05	0.128
	Cond.:Group	1	42	0.444	0.01	0.509	0.238	0.01	0.628	2.510	0.06	0.121	0.224	0.01	0.638
**EDA (left)**	Condition	1	44	0.271	0.01	0.606	0.391	0.01	0.535	0.005	<0.01	0.944	9.220	0.20	**0.004***
	Group	1	44	1.089	0.03	0.303	0.983	0.03	0.328	1.160	0.03	0.288	2.001	0.05	0.165
	Cond.:Group	1	44	0.028	<0.01	0.867	0.361	0.01	0.552	1.155	0.03	0.289	0.231	0.01	0.633
**EDA (right)**	Condition	1	44	0.688	0.02	0.412	0.845	0.02	0.364	0.625	0.02	0.434	0.247	0.01	0.622
	Group	1	44	0.196	0.01	0.661	0.015	<0.01	0.903	0.297	0.01	0.589	4.300	0.10	**0.045***
	Cond.:Group	1	44	0.903	0.02	0.348	3.255	0.08	0.079	0.192	0.01	0.664	<0.001	<0.01	0.983
**HR**	Condition	1	44	6.988	0.14	**0.011***	2.505	0.06	0.121	9.694	0.19	**0.003****	0.001	<0.01	0.979
	Group	1	44	9.311	0.18	**0.004****	1.367	0.03	0.249	7.847	0.16	**0.008***	0.384	0.01	0.539
	Cond.:Group	1	44	0.550	0.01	0.462	0.151	<0.01	0.699	1.555	0.04	0.219	1.702	0.04	0.199
**MAP**	Condition	1	44	0.726	0.02	0.399	0.578	0.01	0.451	0.469	0.01	0.497	0.452	0.01	0.505
	Group	1	44	0.757	0.02	0.389	0.497	0.01	0.485	3.271	0.07	0.077	1.599	0.04	0.213
	Cond.:Group	1	44	0.844	0.02	0.363	0.044	<0.01	0.835	2.117	0.05	0.153	0.268	0.01	0.607
**PP**	Condition	1	42	0.258	0.01	0.614	0.016	<0.01	0.900	1.522	0.03	0.224	0.086	<0.01	0.771
	Group	1	42	0.528	0.01	0.471	0.131	<0.01	0.719	0.007	<0.01	0.932	0.816	0.02	0.372
	Cond.:Group	1	42	0.156	<0.01	0.695	0.718	0.02	0.402	0.001	<0.01	0.982	0.089	<0.01	0.766
**SpO_2**	Condition	1	42	1.139	0.03	0.292	2.212	0.05	0.144	0.197	<0.01	0.660	2.836	0.06	0.099
	Group	1	42	0.720	0.02	0.401	0.390	0.01	0.536	1.296	0.03	0.261	0.928	0.02	0.341
	Cond.:Group	1	42	0.316	0.01	0.577	1.505	0.03	0.226	1.082	0.02	0.304	0.147	<0.01	0.703
**SBP**	Condition	1	44	0.216	<0.01	0.644	0.046	<0.01	0.831	4.057	0.08	0.050	0.224	0.01	0.638
	Group	1	44	0.103	<0.01	0.750	0.002	<0.01	0.968	0.367	0.01	0.548	5.327	0.11	**0.026***
	Cond.:Group	1	44	0.258	0.01	0.614	0.018	<0.01	0.893	0.638	0.01	0.429	0.607	0.01	0.440
**Temp (left)**	Condition	1	44	7.360	0.16	**0.010****	1.096	0.03	0.302	11.969	0.24	**0.001****	3.007	0.07	0.091
	Group	1	44	2.622	0.06	0.114	0.936	0.02	0.339	0.241	0.01	0.626	6.447	0.15	**0.015***
	Cond.:Group	1	44	0.277	0.01	0.602	0.037	<0.01	0.848	1.452	0.04	0.236	1.042	0.03	0.314
**Temp (right)**	Condition	1	44	4.464	0.11	**0.041***	0.277	0.01	0.602	0.115	<0.01	0.736	3.118	0.08	0.085
	Group	1	44	0.135	<0.01	0.715	0.015	<0.01	0.903	2.206	0.05	0.146	0.049	<0.01	0.827
	Cond.:Group	1	44	0.174	<0.01	0.679	0.019	<0.01	0.892	<0.001	<0.01	0.997	4.994	0.12	**0.031***

**p < 0.05; ** significant p-values after the FDR correction*

Table *S3* *post-hoc* statistics of the significant interaction results between the factor condition and group. The contrasts were conducted in R with the *emmeans* library.

Signal	Frequency band	Condition pairwise	Group pairwise	estimate	SE	df	*t*.ratio	*p*.value
$[O_2Hb]$ (left, raw)	LF1	B - T	1 - 2	-0.078	0.026	44	-3.013	0.0043
$[O_2Hb]$ (left+right, raw)	LF1	B - T	1 - 2	-0.061	0.027	44	-2.289	0.0269
[tHb] (left, raw)	LF1	B - T	1 - 2	-0.053	0.023	44	-2.279	0.0276
$[O_2Hb]$ (left)	VLF	B - T	1 - 2	-0.0245	0.0362	44	-0.677	0.5022
[tHb] (left, raw)	VLF	B - T	1 - 2	-0.0637	0.0328	44	-1.940	0.0588
[tHb] (left+right)	VLF	B - T	1 - 2	-0.0885	0.0401	44	-2.208	0.0325
$[O_2Hb]$ (left, raw)	LF2	B - T	1 - 2	-0.0548	0.0262	44	-2.095	0.0420
Temp (right)	HR	B - T	1 - 2	-0.0274	0.0228	38	-1.200	0.2376

Table S4 Wilcoxon rank sum test results of fNIRS coherence coupling analysis for LF1 (0.015–0.15 Hz), LF2 (0.08–0.15 Hz), VLF (0.002–0.08 Hz) and HR (1–2 Hz) frequency band. In the table are listed the absolute value of the Z statistic (|Z|), the difference of the median between the 24 real pairs coherence and the 2400 random pairs coherence, and the *p*-values (*p*). A single asterisk indicates $p < 0.05$, while the double asterisks indicate that the p-values are significant after the false discovery rate correction. All the significant results are marked in bold typeface, where a single asterisk indicates $p < 0.05$ and the double asterisks indicate that the *p*-values are significant after the FDR correction.

Signal	Condition	LF1 band			LF2 band			VLF band			HR band		
		**|*Z*|**	**ΔMed**	***p***	**|*Z*|**	**ΔMed**	***p***	**|*Z*|**	**ΔMed**	***p***	**|*Z*|**	**ΔMed**	***p***
**[HHb] (left)**	Eyes closed	2.89	0.01	**<0.001****	2.34	0.03	**0.019***	2.21	0.01	0.027	0.33	<0.01	0.744
	Eye contact	5.10	0.04	**<0.001****	3.59	0.03	**<0.001****	4.23	0.04	**<0.001****	0.50	<0.01	0.617
**[HHb] (left, raw)**	Eyes closed	1.66	0.02	0.096	1.94	0.02	0.052	0.94	<0.01	0.348	1.40	0.02	0.163
	Eye contact	2.83	0.02	**<0.001****	2.40	0.02	**0.016****	2.47	0.03	**0.013****	2.21	0.03	**0.027***
**[HHb] (right)**	Eyes closed	1.63	0.01	0.104	0.69	<0.01	0.487	0.69	<0.01	0.487	1.21	0.02	0.226
	Eye contact	3.22	0.02	**0.001****	2.14	0.01	**0.033***	3.27	0.04	**0.001****	1.18	0.02	0.237
**[HHb] (right,raw)**	Eyes closed	2.12	0.02	**0.034***	1.76	0.01	0.078	1.75	0.02	0.081	1.14	0.01	0.254
	Eye contact	2.06	0.01	**0.039***	2.08	0.03	**0.037***	2.14	0.02	**0.032***	1.16	<0.01	0.247
**[HHb] (left+right)**	Eyes closed	2.83	0.02	**<0.001****	2.42	0.03	**0.015****	1.79	0.01	0.074	0.58	<0.01	0.559
	Eye contact	4.63	0.03	**<0.001****	2.62	0.02	**0.009****	4.1	0.03	**<0.001****	1.08	0.01	0.279
**[HHb] (left +right, raw)**	Eyes closed	1.72	0.02	0.086	1.54	0.03	0.122	0.83	0.01	0.409	0.97	0.02	0.333
	Eye contact	3.08	0.03	**0.002***	2.42	0.03	**0.015***	3.50	0.04	**<0.001****	1.77	0.02	0.077
**[O_2Hb] (left)**	Eyes closed	1.61	0.01	0.107	1.64	0.02	0.101	0.54	<0.01	0.587	0.52	<0.01	0.605
	Eye contact	3.69	0.03	**<0.001****	2.48	0.02	**0.013****	3.71	0.03	**<0.001****	0.37	<0.01	0.714
**[O_2Hb] (left, raw)**	Eyes closed	1.64	0.01	0.101	0.90	<0.01	0.366	0.74	0.02	0.462	0.16	<0.01	0.869
	Eye contact	0.78	<0.01	0.437	0.45	0.01	0.654	1.58	0.01	0.114	0.74	0.04	0.458
**[O_2Hb] (right)**	Eyes closed	2.27	0.02	**0.023***	2.16	0.02	**0.031***	0.95	0.01	0.340	1.14	0.03	0.256
	Eye contact	3.89	0.03	**<0.001****	3.61	0.03	**<0.001****	3.23	0.03	**0.001****	0.26	<0.01	0.795
**[O_2Hb] (right, raw)**	Eyes closed	1.05	0.01	0.294	0.81	<0.01	0.420	0.50	0.01	0.619	0.18	-0.03	0.854
	Eye contact	1.53	0.02	0.126	0.64	0.02	0.522	1.93	0.01	0.054	0.72	0.03	0.470
**[O_2Hb] (left + right)**	Eyes closed	2.08	0.01	**0.037***	2.86	0.03	**0.004****	0.69	0.01	0.490	0.80	0.01	0.424
	Eye contact	4.17	0.03	**<0.001****	3.87	0.04	**<0.001****	3.35	0.04	**<0.001****	0.04	0.01	0.971

**[O_2Hb] (left + right, raw)**	Eyes closed	1.43	0.01	0.153	1.17	<0.01	0.241	0.56	0.01	0.574	0.11	-0.01	0.915
	Eye contact	0.94	0.01	0.347	0.37	0.01	0.712	1.90	0.02	0.057	0.77	0.03	0.440
**[tHb] (left)**	Eyes closed	0.60	0.01	0.550	1.50	0.02	0.133	0.54	-0.01	0.586	0.34	<0.01	0.736
	Eye contact	3.08	0.03	**<0.001****	3.29	0.02	**0.001****	3.11	0.03	**0.002****	0.15	-0.02	0.884
**[tHb] (left, raw)**	Eyes closed	2.00	0.02	**0.045***	0.23	0.01	0.816	1.55	0.02	0.122	0.05	-0.02	0.960
	Eye contact	0.99	0.01	0.323	0.71	0.01	0.476	1.30	0.01	0.195	0.81	0.04	0.419
**[tHb] (right)**	Eyes closed	0.87	0.01	0.386	0.95	0.01	0.343	0.86	0.01	0.390	0.58	<0.01	0.565
	Eye contact	4.01	0.03	**<0.001****	3.84	0.04	**<0.001****	3.57	0.03	**<0.001****	0.34	0.01	0.730
**[tHb] (right,raw)**	Eyes closed	1.43	0.03	0.154	0.22	<0.01	0.826	1.58	0.03	0.115	0.02	-0.04	0.982
	Eye contact	1.44	0.02	0.149	0.50	0.02	0.617	2.06	0.02	**0.039***	0.62	0.02	0.532
**[tHb] (left + right)**	Eyes closed	0.66	<0.01	0.511	1.71	0.02	0.087	0.67	<0.01	0.500	0.46	<0.01	0.643
	Eye contact	4.13	0.03	**<0.001****	3.66	0.04	**<0.001****	3.58	0.04	**<0.001****	0.03	<0.01	0.973
**[tHb](left+ right,raw)**	Eyes closed	1.86	0.02	0.063	0.38	<0.01	0.703	1.58	0.02	0.115	0.08	-0.04	0.934
	Eye contact	1.33	0.01	0.182	0.45	-0.01	0.652	2.15	0.02	0.031	0.65	0.03	0.513

**p < 0.05; ** significant p-values after the FDR correction.*

Table S5 Wilcoxon rank sum test results of systemic physiology coherence coupling analysis.

Signal	Condition	LF1 band			LF2 band			VLF band			HR band		
		|*Z*|	ΔMed	*p*	|*Z*|	ΔMed	*p*	|*Z*|	ΔMed	*p*	|*Z*|	ΔMed	*p*
$P_{ET}CO_2$	Eyes closed	1.16	<0.01	0.247	0.60	<0.01	0.546	1.44	<0.01	0.150	*NA*	*NA*	*NA*
	Eye contact	0.74	<0.01	0.458	0.26	<0.01	0.793	0.27	<0.01	0.785	*NA*	*NA*	*NA*
DBP	Eyes closed	0.57	<0.01	0.569	0.55	-0.01	0.581	0.90	<0.01	0.370	0.14	-0.01	0.888
	Eye contact	1.77	0.01	0.077	0.09	-0.01	0.930	2.08	0.02	**0.037***	0.40	0.01	0.692
EDA (left)	Eyes closed	1.68	0.02	0.093	0.68	<0.01	0.499	2.55	0.03	**0.011***	0.83	-0.01	0.407
	Eye contact	2.15	0.03	**0.032***	2.02	0.03	**0.044***	2.02	0.03	**0.043***	0.85	0.01	0.397
EDA (right)	Eyes closed	0.26	0.01	0.793	0.08	<0.01	0.938	0.17	<0.01	0.865	0.19	<0.01	0.850
	Eye contact	1.80	0.01	0.072	1.46	0.01	0.145	2.34	0.03	**0.019***	0.12	<0.01	0.906
HR	Eyes closed	1.07	0.01	0.287	0.08	<0.01	0.936	2.24	0.02	**0.025***	1.16	<0.01	0.244
	Eye contact	2.26	0.02	**0.024***	2.67	0.03	**0.008***	2.45	0.01	**0.014***	0.76	0.01	0.447
MAP	Eyes closed	0.59	<0.01	0.558	0.77	-0.01	0.440	0.16	<0.01	0.871	0.64	-0.01	0.520
	Eye contact	0.50	<0.01	0.619	0.05	<0.01	0.957	1.19	<0.01	0.235	0.08	0.01	0.935
PP	Eyes closed	0.67	-0.01	0.502	0.08	<0.01	0.934	1.14	-0.01	0.253	0.10	0.01	0.917
	Eye contact	0.55	<0.01	0.582	0.87	-0.01	0.387	1.30	0.01	0.194	0.58	<0.01	0.561
SpO_2	Eyes closed	1.06	-0.01	0.288	0.02	-0.01	0.982	1.94	-0.03	0.052	0.18	<0.01	0.860
	Eye contact	0.25	<0.01	0.805	1.42	0.03	0.155	1.18	-0.01	0.239	0.02	<0.01	0.986
SBP	Eyes closed	0.81	-0.01	0.420	0.64	<0.01	0.521	1.06	-0.01	0.291	0.46	0.01	0.644
	Eye contact	0.04	<0.01	0.964	0.82	-0.01	0.412	1.16	0.02	0.245	0.34	<0.01	0.733
Temp (left)	Eyes closed	0.81	0.01	0.419	0.70	<0.01	0.484	0.19	<0.01	0.849	0.24	<0.01	0.810
	Eye contact	0.66	<0.01	0.508	0.42	<0.01	0.671	0.82	<0.01	0.411	0.74	<0.01	0.459
Temp (right)	Eyes closed	0.75	<0.01	0.454	0.53	<0.01	0.599	0.15	-0.01	0.879	0.33	<0.01	0.743
	Eye contact	1.82	-0.02	0.068	1.04	<0.01	0.298	0.98	<0.01	0.326	0.01	<0.01	0.995

**p < 0.05; ** significant p-values after the FDR correction.*

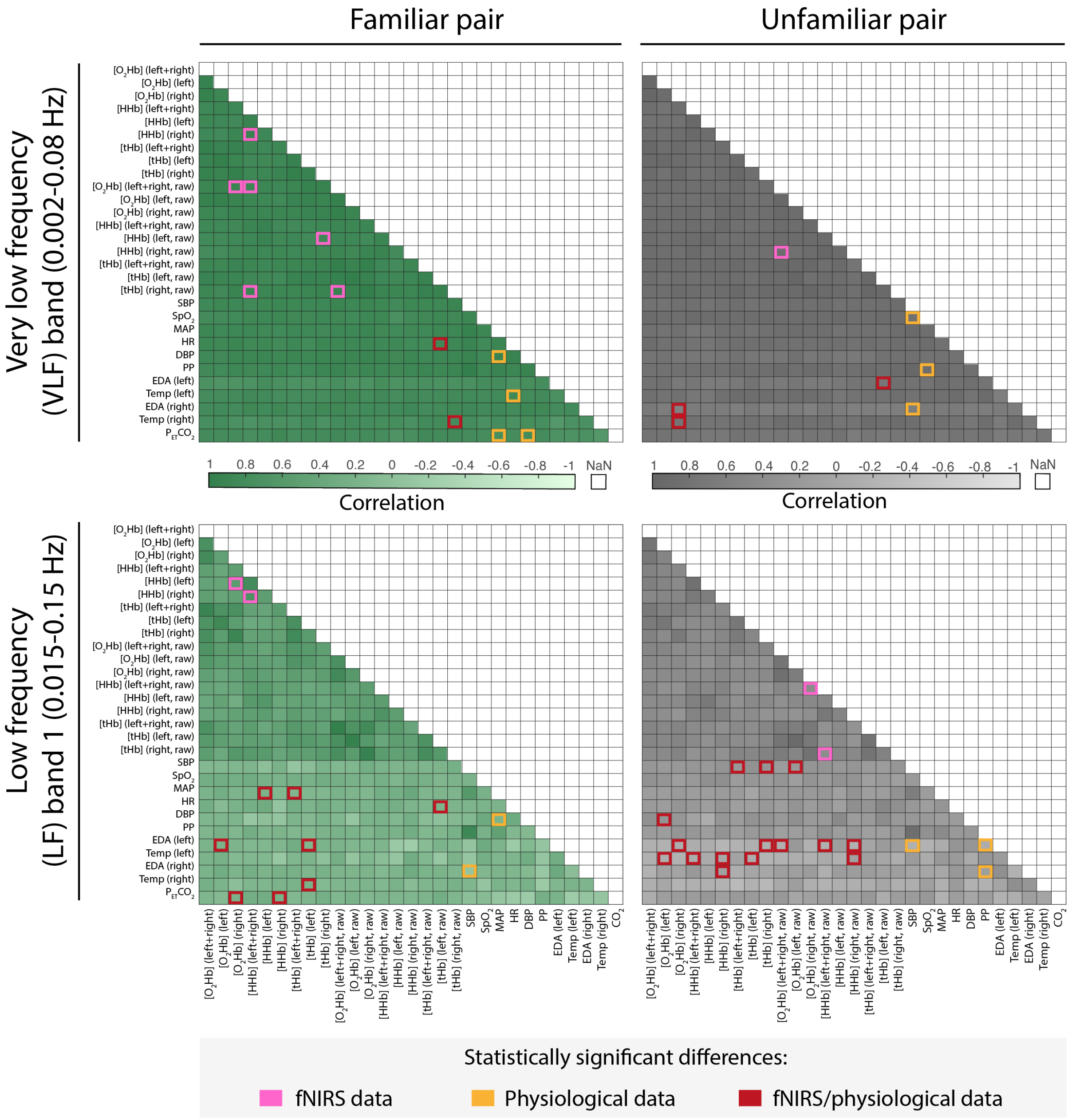

Fig. S1 Group averages of the difference between the eyes-open and the eyes-closed and condition correlation matrices comparing the two groups (familiar pair and unfamiliar pair) in the VLF band (top left and top right, respectively) and LF1 band (bottom left and bottom right, respectively). In the two frequency bands, a marked matrix value indicates a significant difference and a higher median of the difference of distributions (eyes-open correlation distribution and eyes-closed correlation distribution) than the other group of the respective frequency band. The statistical differences occurred only before performing the FDR correction.

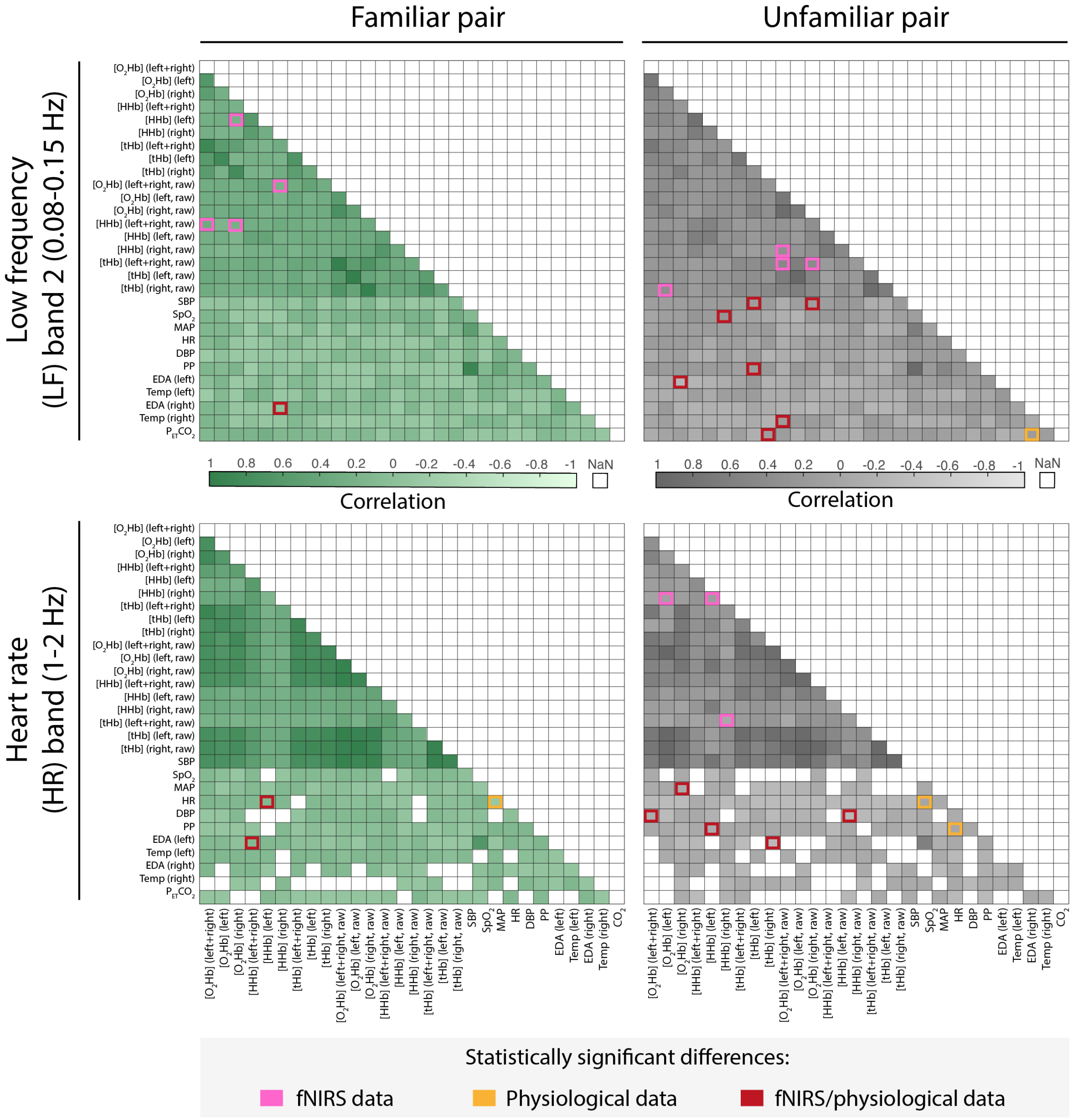

Fig. S2 Group averages of the difference between the eyes-open and the eyes-closed and condition correlation matrices comparing the two groups (familiar pair and unfamiliar pair) in the LF2 band (top left and top right, respectively) and HR frequency band (bottom left and bottom right, respectively). In the two frequency bands, a marked matrix value indicates a significant difference and a higher median of the difference of distributions (eyes-open correlation distribution and eyes-closed correlation distribution) than the other group of the respective frequency band. The statistical differences occurred only before performing the FDR correction.
